# Intellectual functioning and behavioural features associated with mosaicism in fragile X syndrome

**DOI:** 10.1186/s11689-019-9288-7

**Published:** 2019-12-26

**Authors:** Emma K. Baker, Marta Arpone, Solange Aliaga Vera, Lesley Bretherton, Alexandra Ure, Claudine M. Kraan, Minh Bui, Ling Ling, David Francis, Matthew F. Hunter, Justine Elliott, Carolyn Rogers, Michael J. Field, Jonathan Cohen, Lorena Santa Maria, Victor Faundes, Bianca Curotto, Paulina Morales, Cesar Trigo, Isabel Salas, Angelica M. Alliende, David J. Amor, David E. Godler

**Affiliations:** 1 Diagnosis and Development, Murdoch Children’s Research Institute, Royal Children’s Hospital, Melbourne, VIC Australia; 2 Faculty of Medicine, Dentistry and Health Sciences, Department of Paediatrics, University of Melbourne, Parkville, VIC Australia; 3 School of Psychology and Public Health, La Trobe University, Bundoora, VIC Australia; 4 Brain and Mind, Murdoch Children’s Research Institute, Royal Children’s Hospital, Melbourne, VIC Australia; 5 Neurodisability and Rehabilitation, Murdoch Children’s Research Institute, Royal Children’s Hospital, Melbourne, VIC Australia; 6 Royal Children’s Hospital, Melbourne, VIC Australia; 7 Department of Pediatrics, Monash University, Clayton, VIC Australia; 8 Centre for Epidemiology and Biostatistics, Melbourne School of Population and Global Health, University of Melbourne, Carlton, VIC Australia; 9 Victorian Clinical Genetics Services and Murdoch Children’s Research Institute, Royal Children’s Hospital, Melbourne, VIC Australia; 10 Monash Genetics, Monash Health, Melbourne, VIC Australia; 11 Genetics of Learning Disability Service, Hunter Genetics, Waratah, NSW Australia; 12 Fragile X Alliance Inc, Centre for Developmental Disability Health Victoria, Monash University, North Caulfield, Clayton, VIC Australia; 13 Laboratory of Molecular Cytogenetics, Department of Genetics and Metabolic Diseases, Institute of Nutrition and Food Technology (INTA), University of Chile, Santiago, Chile

**Keywords:** Fragile X syndrome, Mosaicism, Autism spectrum disorder, Intellectual functioning, Behaviour, Phenotype

## Abstract

**Background:**

Fragile X syndrome (FXS) is a common cause of intellectual disability and autism spectrum disorder (ASD) usually associated with a CGG expansion, termed full mutation (FM: CGG ≥ 200), increased DNA methylation of the *FMR1* promoter and silencing of the gene. Mosaicism for presence of cells with either methylated FM or smaller unmethylated pre-mutation (PM: CGG 55–199) alleles in the same individual have been associated with better cognitive functioning. This study compares age- and sex-matched FM-only and PM/FM mosaic individuals on intellectual functioning, ASD features and maladaptive behaviours.

**Methods:**

This study comprised a large international cohort of 126 male and female participants with FXS (aged 1.15 to 43.17 years) separated into FM-only and PM/FM mosaic groups (90 males, 77.8% FM-only; 36 females, 77.8% FM-only). Intellectual functioning was assessed with age appropriate developmental or intelligence tests. The Autism Diagnostic Observation Schedule-2nd Edition was used to examine ASD features while the Aberrant Behavior Checklist-Community assessed maladaptive behaviours.

**Results:**

Comparing males and females (FM-only + PM/FM mosaic), males had poorer intellectual functioning on all domains (*p* < 0.0001). Although females had less ASD features and less parent-reported maladaptive behaviours, these differences were no longer significant after controlling for intellectual functioning. Participants with PM/FM mosaicism, regardless of sex, presented with better intellectual functioning and less maladaptive behaviours compared with their age- and sex-matched FM-only counterparts (*p* < 0.05). ASD features were similar between FM-only and PM/FM mosaics within each sex, after controlling for overall intellectual functioning.

**Conclusions:**

Males with FXS had significantly lower intellectual functioning than females with FXS. However, there were no significant differences in ASD features and maladaptive behaviours, after controlling for intellectual functioning, independent of the presence or absence of mosaicism. This suggests that interventions that primarily target cognitive abilities may in turn reduce the severity of maladaptive behaviours including ASD features in FXS.

## Background

Fragile X syndrome (FXS) is a common single-gene cause of inherited intellectual disability (ID) and autism spectrum disorder (ASD), with current prevalence estimates of 1:4000 and 1:8000 males and females, respectively [1]. The clinical phenotype of FXS is heterogeneous, affected by age, sex and genetic and epigenetic factors (e.g. X chromosome inactivation in females). The primary cause of FXS is a large trinucleotide CGG expansion (≥ 200 repeats), termed full mutation (FM), in the promoter region of the fragile X mental retardation 1 (*FMR1*) gene [2]. FM alleles are associated with DNA methylation (DNAm) changes to the *FMR1* promoter, resulting in decreased transcription [3] and little to no production of the fragile X mental retardation protein (FMRP). FMRP has been shown to regulate genes that have been implicated in ASD [4] and to be important for neurodevelopment [5].

Mosaicism for smaller alleles usually unmethylated, termed pre-mutation (PM 55–199 CGG repeats) in combination with methylated FM alleles has been reported in 34% of males and 10% of females with FXS using methylation-sensitive Southern blot techniques [6, 7]. The phenotype is typically reported to be attenuated in such cases due to the presence of cells with the unmethylated *FMR1* promoter, capable of producing FMRP, though variability is still observed [8]. Nonetheless, the biological mechanisms responsible for the variability in the FXS phenotype especially in females and PM/FM mosaic individuals is not well understood. The contribution of genotype mosaicism on psychological functioning, within and between sexes, may shed light on the underlying biology that contributes towards the cognitive, emotional and behavioural phenotype in males and females with FXS.

Females with FM are typically reported to be less affected than males due to the presence of the second normal size (< 44 CGG) *FMR1* allele located on the active X chromosome in some cells [9]. The vast majority of males with FXS typically present with ID [7, 8, 10] with intellectual quotients (IQs) below 70. Their clinical profile is also characterised by attention and executive functioning difficulties, anxiety, social withdrawal, hyperactivity, impulsivity and ASD features [11]. The phenotype in females with a FM is less predictable, which in part may be due to an underrepresentation of females in the FXS literature [12]. A wide range of intellectual abilities has been associated with the female FXS phenotype, with IQ ranging from the *extremely low* to the *superior* range [13, 14]. Females with FXS can also present with a variety of behavioural and emotional problems, including attention difficulties, anxiety, withdrawn behaviours and ASD features with associated impacts on outcomes [12, 15, 16]. In particular, elevated rates of socially avoidant behaviours and repetitive and restricted behaviours (RRB) have been noted in females with FXS compared with non-FXS females [17, 18].

Males with mosaic FXS have been generally reported to have better intellectual functioning than their FM-only counterparts [19]. However, studies comparing rates of ASD between FM-only and mosaic cases are somewhat limited, as these have primarily focused on males, not both sexes. A study of boys with FXS (mean age 56.6 ± 13.7 months) demonstrated that 18 of 41 (43.9%) participants with FM and 6 of 15 (40%) participants with mosaicism (DNAm + PM/FM) met DSM-IV criteria for ASD [20]. In contrast, in an earlier study [21], there was a trend for ASD to be more likely diagnosed in FM-only males (44%; *n* = 18) in comparison with males with mosaic FXS (18%; *n* = 28).

In females, Rousseau and colleagues [8] did not identify a significant difference in the distribution of degrees of intellectual impairment between females with FM-only and PM/FM mosaicism. Remarkably, intellectual functioning was not formally assessed and was assigned based on clinical judgement. Thus, further research comparing intellectual functioning and behavioural features between females with FM-only and PM/FM mosaicism with validated standardised measures is an important gap that needs to be addressed.

Chronological age has also been associated with the clinical phenotype, with an apparent cognitive decline in males with FXS. However, a more recent large prospective longitudinal study [14] identified a widening gap in cognitive performance between participants with FXS relative to the normative sample in childhood, but with a stabilisation or narrowing of this gap during adolescence. For behavioural profiles, increasing chronological age has been associated with decreases in scores on the Aberrant Behavior Checklist-Community (ABC-C [22]) in both males and females with FXS [23]. While ASD prevalence and symptom severity was reported to increase over time in individuals with FXS [24].

One of the most consistently reported behavioural characteristics of FXS is the presence of ASD features, with up to 90% of males and 50% of females demonstrating some symptoms associated with ASD [11, 25]. However, there is contention as to whether comorbid ASD in FXS is a true phenomenon, or indeed that ASD and FXS represent categorically and qualitatively distinct disorders [26, 27]. Reported prevalence of ASD in FXS varies between studies depending on the assessment tools and diagnostic criteria that are used. In a systematic review and meta-analysis of ASD phenomenology in various genetic disorders, approximately 22% of individuals with FXS met criteria for ASD [28]. The prevalence estimate rose to 30% when only males with FXS were included. The presence of seizures in FXS has also been linked to the presence of ASD. Utilising the fragile X clinical and research consortium database prevalence rates for seizures in males and females with FXS are 12.1% and 3.2%, respectively, with estimates for males and females with FXS + ASD being 16% [29].

The differences in prevalence estimates of ASD observed between males and females with FXS have been attributed to the more favourable genetic and biochemical functioning of females (i.e. the presence of a normal *FMR1* allele on the active X chromosome expressing FMRP) [27]. Moreover, the more severe intellectual impairments in males with FXS have been associated with more ASD features [27]. Furthermore, idiopathic ASD is more common in males [30, 31]. Several biological explanations for the male preponderance in ASD have been proposed, including male vulnerability, female protection and the possibility of ascertainment bias and associated female under diagnosis (see Ferri et al. [30] for a review) particularly for those without an ID [32]. To this end, Ratto and colleagues [33] undertook item-level analysis on the ADOS-2 to determine the specific profiles of males and females on this measure. The authors demonstrated that while IQ-matched males and females had similar overall calibrated severity scores (CSS) and that item-level differences did indeed emerge, none of these differences survived corrections for multiple testing. In contrast, similar analyses comparing males and females with FXS have not previously been undertaken.

FMRP levels in blood have been shown to be associated with developmental and intellectual functioning levels in males with FXS, with lower levels of FMRP corresponding to more severe cognitive impairments [10, 34, 35]. However, similar relationships have not been shown with behavioural symptoms [36, 37]. Specifically, FMRP levels in peripheral tissues (that may not accurately reflect brain specific variability in FMRP) were not associated with parent reports of behavioural problems, adaptive behaviours or ASD features in a large sample of male and female children (6 to 17 years) with FXS [36]. Moreover, in studies using objective assessments including the Autism Diagnostic Observation Schedule-Generic (ADOS-G), the associations between FMRP and ASD symptom severity were lost after controlling for IQ [37]. Similarly, McDuffie and colleagues found that FMRP did not account for unique variance in reciprocal social interaction, communication and restricted interests, and repetitive behavior domain scores on the Autism Diagnostic Interview-Revised (ADI-R) [38], over and above the contribution of nonverbal IQ, though this study included both males and females in the same cohort, allowing greater data dispersion. More recently, FM-only males with incomplete silencing of *FMR1* mRNA have been reported to have significantly greater severity of ASD features (ADOS-2 calibrated severity scores [CSS]) compared with FM-only males with complete silencing. This suggests that two reciprocal mechanisms, RNA toxicity and FMRP deficiency, may contribute to ID and ASD features in those males with FXS and the *FMR1* gene incompletely silenced [39], though replication of this study in larger independent cohorts is required.

Female-only studies are few, with inconsistent findings between FMRP levels and the clinical phenotype. Some studies have demonstrated associations with intellectual functioning [35, 40] and the behavioural phenotype [41, 42], while others have demonstrated no associations between these constructs and FMRP levels in females with FXS [26, 41, 43–45]. Inconsistent findings in females may be due to the age ranges used in each of the studies. Specifically, Lightbody and colleagues [45] demonstrated that chronological age was associated with executive functioning abilities in females with FXS, but not FMRP levels in blood.

While previous research has comprehensively documented the intellectual functioning phenotypes in males and females with FXS, and to some extent PM/FM mosaic males, there is little understanding of how ASD features and maladaptive behaviours differ in males and females with FXS, as well as between sex-matched FM-only and PM/FM mosaic individuals. Understanding predictors of the behavioural phenotype in both males and females with FXS will assist in determining potential targets for interventions that can assist in improving outcomes. Thus, this study characterises a large international FXS cohort in order to: (i) describe and compare IQ, ASD features and maladaptive behaviours between sexes and between allelic classes within each sex; and (ii) evaluate if the variability of behavioural problems within each sex and between allelic classes is explained by the variability in intellectual functioning.

## Methods

### Participants

This study used a large international FXS cohort recruited from Australia and Chile. Ninety males (51.1% Australian) and 36 females (75% Australian) participated in the study. Male participants were aged between 1.15 and 43.17 years (*M* = 12.06, SD = 9.82) while female participants were aged between 1.71 and 34.13 years (*M* = 10.90, SD = 9.23). The cohort of individuals encompasses a wide age range to enable a sufficient number of females to be included in the current study. In the male cohort, 70 (77.8%) participants were FM-only and 20 (22.2%) were PM/FM mosaic. In the female cohort, 28 (77.8%) were FM-only and 8 (22.2%) were PM/FM mosaic.

Australian participants were recruited via Victorian Clinical Genetics Services, Monash Genetics and Hunter Genetics, and support organisations including the Fragile X Association of Australia and Fragile X Alliance Inc. In Chile, participants were recruited primarily through Molecular and Cytogenetics at INTA, University of Chile. Individuals were excluded from the study if they had any other significant medical conditions (e.g. stroke, head trauma), any other genetic conditions of known clinical significance, and if they had inadequately controlled seizures. Parents completed a developmental and medical history questionnaire to obtain information regarding ethnicity, medication use and other previous or existing diagnoses (e.g. lifetime prevalence of seizures).

### Materials

#### Genetic testing

For the Chilean and Australian participants, inclusion criteria into this study included a confirmed diagnosis of FXS using methylation SB and CGG PCR sizing. Specifically, for all participants, routine FXS testing involved first-line PCR-based assessment of CGG repeat size (with precision of ± one repeat) using a validated PCR amplification assay, with the upper limit of detection of 330 CGG repeats for the Chilean cohort [46] and 170 CGG repeats for the Australian cohort [47]. All samples that showed a CGG size in the PM range or that failed to show a PCR product for males or had a single peak by PCR for females were referred for second-line testing involving methylation-sensitive SB analysis, performed as described [48, 49]. If there were any discrepancies between SB performed between the Australian and Chilean sites, AmplideX long-range PCR was undertaken, as per manufacturer’s instructions [50] on blood or saliva DNA collected as previously described [49]. Original diagnostic reports were also obtained for all Australian participants to confirm diagnosis.

#### Assessment of intellectual functioning

Participants were assessed with one of the following standardised assessments depending on their age and country of residence: the Mullen Scales of Early Learning (MSEL; Australian children aged < 3 years) [51], the Wechsler Preschool and Primary Scale of Intelligence-3rd Australian [52] and  Mexican [53] Editions (WPPSI-III; children aged 3–6 years), the Wechsler Scale of Intelligence-4th Edition Australian (WISC-IV; Australian children aged 7–16 years) [54], Wechsler Scale of Intelligence-3rd Edition Chilean version [55] (WISC-III; Chilean children aged 7-16 years) or the Wechsler Adult Intelligence Scale-4th Australian [56] and Chilean [57] Editions (WAIS-IV; ≥ 17 years). As the MSEL does not provide separate verbal IQ (VIQ) and performance IQ (PIQ) scores, these were derived as described by Richler and colleagues [58]. The Early Learning Composite (ELC) was used as a proxy for Full Scale IQ (FSIQ) [59]. The breakdown of which intellectual functioning assessments were completed, by sex and allelic classification, is presented in Additional file 1: Table S1 (see also Additional file 1: Note S1).

Given the tendency for individuals with FXS, particularly males, to have scores on standardised assessments at floor level, we used corrected IQ scores [60]. Corrected IQ scores were derived using the Whitaker and Gordon method [61]. Briefly, all scaled scores (SS) of 1 were re-examined. In order to calculate corrected index scores (e.g. cVIQ, cFSIQ), the best fit equations between raw scores and SS, and subsequently between sum of scaled scores (SSS) and index scores were determined by using the raw score to SS and the SSS to index scores conversion tables available in the published test manuals. For an example of the application of this technique, see the supplemental material of Arpone et al. [60]. This method reduces loss of data due to invalid scores and has been shown to result in a normal distribution of scores [60]. A caveat to this method in the current sample is that the WISC-III (Chilean version) incorporates more sub-tests in the verbal and performance indexes than the other Wechsler scales used in this study. Sub-tests that are included in the working memory and processing speed indices of the other Wechsler scales (i.e. WISC-IV and WAIS-IV) are included in the VIQ and PIQ, respectively, of the WISC-III. Moreover, while VIQ, PIQ and FSIQ scores can be derived from the MSEL, the assessment is qualitatively different to the Wechsler scales and correction of these scores has not previously been undertaken. Therefore, in the supplementary materials, we also present the analyses using the following: (i) standard scores and (ii) corrected scores with participants assessed with the MSEL and WISC-III removed.

#### Assessment of autism spectrum disorder features

The Autism Diagnostic Observation Schedule-2nd Edition (ADOS-2) [62] was used to assess ASD features. Participants are assessed with one of five modules depending on their age and language abilities. For modules 1–4, the ADOS-2 provides three specific classifications: non-spectrum, autism spectrum and autism. For the toddler module, classifications are based on range of concern: little-to-none, mild-to-moderate and moderate-to-severe. Those falling in the little-to-none were classified as non-spectrum, while those in the latter two ranges were classified as autism spectrum and autism, respectively. Separate CSS based on the overall, social affect (SA) and restricted and repetitive behaviour (RRB) scores are also derived for each module [63–65]. The breakdown of modules completed, by sex and allelic classification, is presented in Additional file 1: Table S1. ADOS-2 assessments and coding were conducted by research members who had undertaken ADOS-2 for research training and had demonstrated > 80% coding reliability across all five modules. ADOS-2 assessments were video recorded, with consent, and a proportion were re-coded with adequate reliability (see also Additional file 1: Note S2).

#### Assessment of maladaptive behaviours

Maladaptive behaviours were assessed in both cohorts using the Aberrant Behavior Checklist-Community (ABC-C) [22]. The ABC-C is a parent-report questionnaire that is commonly used to assess maladaptive behaviours in children with ID. In contrast to the original ABC-C, Sansone and colleagues [66] found a six-factor structure when used in a sample of 630 individuals with FXS. The inappropriate speech domain remained unchanged while the remaining four subscales were modified. A new social avoidance subscale was derived for the sixth factor. These new subscales have demonstrated good internal consistency previously (Cronbach’s *α* = .80–.94 [66]) and in the current sample (Cronbach’s *α* = .82–.94). Kerr et al. [67] also developed a utility index (UI) to determine FXS quality of life based on the ABC-C. Higher scores on the ABC-C indicate greater maladaptive behaviours, while lower UI scores indicate lower/poorer quality of life. Two PM/FM mosaic adult females attended their appointment without a parent/carer and therefore, no ABC-C was available for these participants (see Additional file 1: Note S3).

### Procedure

Participants were initially screened for eligibility criteria. Eligible participants were booked for assessment by a member of the research team that would not be involved in assessments, to ensure the allelic classification was blinded to the assessor. In the majority of cases, the ADOS-2 was administered first, followed by the intellectual functioning assessment. Assessments were typically undertaken on the same day by the same assessor. Two participants were assessed over 2 days to alleviate their distress. A third participant was also assessed over 2 days to accommodate a multiplex family where three children were assessed in succession over a 2-day period. In Chile, all assessments were undertaken in the clinic, while in Australia, 75.3% of assessments were completed in a clinic; the remaining assessments were undertaken in the home.

### Statistical analysis

Summary statistics were presented as frequencies and proportion for categorical variables and mean and standard deviation (SD) for continuous variables. Comparisons were performed between: (i) males and females, and (ii) FM-only and PM/FM mosaic groups for both sexes. Comparisons for categorical variables (demographic information and key ADOS-2 items) were carried out using Fisher’s exact test. For continuous variables, comparisons were performed using regression methods, including a between-group term, adjusted for potential confounders, age and country. A non-linear relationship between each corrected intellectual functioning score and age was observed only in males, and therefore, we used semi-parametric regression (see Additional file 1: Note S4 for further details). Additionally, there were significant differences between the two countries on corrected intellectual functioning variables for males, but not females. For females, no significant relationship was observed between corrected intellectual functioning scores and age. Given the wide age range included in the study, where possible analyses were also conducted in stratified age groups, under 13 years and 13 years and over. Details of the specific analyses including which confounders were controlled in each analysis are described in the footnotes of each table.

Comparisons between males and females and allelic classes on key ADOS-2 items were performed by exploring the proportion of individuals who had atypical presentations on each item. Codes of 1, 2 and 3 were combined to indicate an atypical presentation on the specific behaviour, while scores of 0 indicated a typical presentation.

False discovery rate (FDR) was used to adjust for multiple testing. All analyses were carried out using commercial software Stata *version 15* (http://www.stata.com), and *p* values were two-sided with an alpha level of 0.05.

## Results

### Medical history and demographic information comparisons

There were no differences in the parent-reported lifetime prevalence of seizures and ethnicity between FM and PM/FM mosaics within each sex and between sexes (Table 1). Males were more likely to be taking psychoactive mediations compared with females, though medication use did not differ between FM-only and PM/FM mosaic cases within each sex. The majority of individuals reported to have ever experienced seizures, met criteria for ASD on the ADOS-2 and also met criteria for ID (FSIQ < 70; 8 males with FM-only, 1 male with PM/FM mosaicism, 1 female with FM-only). There was only one individual (FM-only male) with parent-reported seizures who did not meet criteria for ASD. The two sexes did not differ on age (*p* = 0.541).

**Table 1:** Demographic information for males and females by allelic classes

	Males			Females			Males vs. females
	FM-only (*n* = 70) (%)	PM/FM mosaic (*n* = 20) (%)	*p*	FM-only (*n* = 28)^a^ (%)	PM/FM mosaic (*n* = 8) (%)	*p*	*p*
Seizures^b^	13.0	5.6	0.680	3.7	0.0	0.999	0.175
Medication							
Medicated	44.3	30.0	0.309	25.9	0.0	0.166	*0.036*
Stimulant	20.0	10.0	0.508	7.4	0.0	0.999	0.097
SSRI	21.4	5.0	0.109	11.1	0.0	0.999	0.271
SNRI	0.0	5.0	0.222	3.7	0.0	0.999	0.483
Benzodiazepines	0.0	5.0	0.222	0.0	0.0	-	0.999
Anti-psychotic	11.4	10.0	0.999	3.7	0.0	0.999	0.180
Anticonvulsant	7.1	5.0	0.999	3.7	0.0	0.999	0.672
Adrenergic alpha agonist	4.3	5.0	0.999	3.7	0.0	0.999	0.999
Melatonin	8.6	0.0	0.333	0.0	0.0	0.999	0.184
Ethnicity^c^							
Australian/European	36.2	55.0	0.407	66.7	50.0	0.219	0.076
Mestizo	46.4	35.0		18.5	50.0		
Other*	17.4	10.0		14.8	0.0		

^a^Demographic information was not available for one female with FM-only

^b^Seizure information was missing for 2 males with FM-only and 1 male with PM/FM mosaicism

^c^Ethinicty was missing for one male with FM-only

*Other ethnicity included Australian Aboriginal, Asian (Southern/South East), Middle Eastern, Polynesian, European (southern/northwest), American Indian, multi-ethnic and not disclosed. All *p* values computed using Fisher’s exact test

### Correlation between ASD features and corrected intellectual functioning

Corrected VIQ and PIQ (cVIQ and cPIQ) were inversely correlated with ASD features (SA CSS and RRB CSS), in males and females (FM-only + PM/FM mosaic), with the exception of cPIQ and SA CSS in females, and cVIQ and RRB CSS in males (Additional file 1: Table S3). The ADOS CSS was also inversely correlated with all corrected intellectual functioning scores in males and females (all *p* < 0.05), except between cWMI in females (*p* = 0.253). Thus, when comparing corrected IQ scores analyses were adjusted for ADOS CSS and similarly when comparing ADOS CSS, analyses were adjusted for corrected IQ scores.

### Comparison of intellectual functioning, ASD features and maladaptive behaviours between males and females

Sixty-three percent of males and 7.1% of females with FXS obtained a standard FSIQ score that was either invalid or at floor level (e.g. FSIQ = 40). The proportions of standard IQ scores that were invalid and at floor level, stratified by sex and allelic class, are reported in Additional file 1: Table S2. A significantly higher proportion of males (93.3%) had an ID (standard FSIQ < 70), compared with females (47.2%; Fisher’s exact test, *p* < 0.001). Males with FXS had significantly poorer corrected IQ scores on all domains compared with females (Table 2). These significant differences were still observed when standard scores were used and when the MSEL and WISC-III scores were removed from the corrected scores (Additional file 1: Tables S4 and S5, respectively).

**Table 2:** Comparison between males and females on corrected intellectual functioning scores, ASD features, and maladaptive behaviours

	Males		Females		*p*
	*n*	Mean ± SD	*n*	Mean ± SD	
Intellectual functioning^1^					
cVIQ	87	45.3 ± 23.6	36	74.1 ± 18.6	< 0.0001*
cPIQ	88	44.7 ± 19.7	36	69.0 ± 16.9	< 0.0001*
cWMI	34	35.8 ± 15.1	13	66.0 ± 18.6	< 0.0001*
cPSI	46	45.1 ± 17.6	22	80.1 ± 20.0	< 0.0001*
cFSIQ	87	33.3 ± 24.2	36	67.8 ± 17.8	< 0.0001*
ASD Features^2^					
ADOS CSS	80	6.54 ± 2.11	32	4.53 ± 2.29	0.090
SA CSS	80	6.24 ± 2.25	32	4.56 ± 2.15	0.253
RRB CSS	80	7.58 ± 1.89	32	6.16 ± 2.64	0.129
Maladaptive behaviours^3^					
Irritability	87	14.1 ± 11.0	34	10.1 ± 11.3	0.523
Lethargy	87	6.22 ± 5.20	34	4.62 ± 5.41	0.829
Stereotypy	87	5.22 ± 4.66	34	2.76 ± 4.31	0.057
Hyperactivity	87	11.3 ± 8.23	34	6.79 ± 7.50	0.071
Inappropriate speech	87	4.45 ± 3.51	34	2.59 ± 2.96	0.724
Social avoidance	87	3.02 ± 2.78	34	2.38 ± 3.21	0.130
ABC total	87	44.3 ± 28.0	34	29.2 ± 29.3	0.306
ABC UI	87	0.64 ± 0.19	34	0.73 ± 0.20	0.415

^1^Semi-parametric regression adjusted for country, age and ADOS CSS

^2^Robust regression adjusted for country and cFSIQ^3^Robust Regression adjusted for cFSIQ and age

*p value remained < 0.05 after adjustment for multiple testing using FDR

A significantly higher proportion of males (90.4%) met ADOS-2 criteria for ASD compared with females (65.6%; Fisher’s exact test, *p* = 0.004). Examining the specific ADOS-2 cutoffs 13 (40.6%) females and 19 males (22.9%) met the autism spectrum/mild-to-moderate concern cutoff, while eight (25.0%) females and 56 (67.5%) males met the autism/moderate-to-severe concern cutoff. However, males and females did not differ significantly on ADOS-2 CSS scores after controlling for country and cFSIQ (Table 2).

Moreover, when the data was stratified according to two age groups (< 13 years and ≥ 13 years), the differences between males and females for corrected IQ scores, ASD features and maladaptive behaviours were similar to those shown in Table 2 (Additional file 1: Tables S6 and S7).

Comparisons between males and females on specific ADOS-2 items showed significant differences on several items including eye contact, shared enjoyment, showing, initiation of joint attention, rapport, sensory behaviours, response to name, amount of social overtures (both to the examiner and caregiver), amount of reciprocal communication and functional and creative play items (Table 3). Similar results were observed for individuals aged < 13 years (Additional file 1: Table S8). However, for the older age group (≥ 13 years), no significant differences between males and females were observed, likely due to the small sample of females (*n* = 8) in this age group reducing the power to detect significant differences (Additional file 1: Table S9).

**Table 3:** Comparison between males and females on key ADOS-2 items

	Males		Females		*p*
	*n*	% atypical	*n*	% atypical	
SA					
Pointing	62	71.0	17	76.5	0.767
Gestures	83	55.4	32	37.5	0.099
Eye contact	83	81.9	32	53.1	0.004*
Facial expressions	83	88.0	32	71.9	0.050
Shared enjoyment	83	49.4	32	18.8	0.003*
Showing	62	77.4	17	35.3	0.002*
Response to joint attention	62	29.0	17	11.8	0.212
Initiation of joint attention	62	71.0	17	29.4	0.004*
Quality of social overtures	83	88.0	32	75.0	0.095
Rapport	83	72.3	32	46.9	0.016*
RRB					
Sensory	83	62.7	32	31.3	0.003*
Mannerisms	83	59.0	32	46.9	0.296
Repetitive and stereotyped behaviours	83	73.5	32	68.8	0.646
Stereotyped language	62	78.7	27	63.0	0.187
Other					
Use of body as tool	39	38.5	9	11.1	0.238
Response to name	62	43.5	17	11.8	0.022*
Social smile	33	51.5	6	16.7	0.190
Amount of social overtures (examiner)	83	72.3	32	37.5	0.001*
Amount of social overtures (caregiver)	48	55.1	16	6.3	0.001*
Amount of reciprocal communication	42	66.7	23	30.4	0.009*
Functional play	62	69.4	17	29.4	0.005*
Imaginative/creative play	83	94.0	31	67.7	0.001*
Self-injury	83	12.0	32	0.0	0.060
Anxiety	83	36.1	31	35.5	0.999

All *p* values computed using Fisher’s exact test

*p value remained < 0.05 after adjusting for multiple testing using FDR

Males and females did not significantly differ on any ABC-C subscales after adjusting for age and cFSIQ (Table 2). When comparing the specific classifications of the UI, a significantly greater proportion of males (47.8%) fell in the very low or low range compared with females (23.5%), *p* = 0.008. Nineteen (55.9%) females were classified as having high or very high quality of life compared with only 24 (26.7%) males. All other individuals fell in the moderate range.

### Comparison of intellectual functioning, ASD features and maladaptive behaviours between males with FM-only and PM/FM mosaicism

Sixty-five (94.2%) males with FM-only and 18 (90.0%) males with PM/FM mosaicism met criteria for ID (*p* = 0.613). However, males with FM-only had statistically significant lower cVIQ, cPSI and cFSIQ compared with males with PM/FM mosaicism (Table 4), though the difference for cVIQ did not survive FDR. When standard IQ scores were used VIQ, WMI and PSI differed significantly between the two groups (Additional file 1: Table S10). When those males assessed with the MSEL and WISC-III were removed from the corrected scores, differences between the male PM/FM mosaic and FM-only groups were no longer significant (Additional file 1: Table S11). When stratified by age, significant differences were observed for males under 13 years for all intellectual functioning scores, except cPIQ after adjusting for multiple testing (Additional file 1: Table S12). However, no significant differences were observed between males with FM-only and PM/FM mosaicism aged 13 years and above (Additional file 1: Table S13).

**Table 4:** Comparison of males by allelic class on corrected intellectual functioning, ASD features and maladaptive behaviours

	FM-only		PM/FM Mosaic		*p*
	*n*	Mean ± SD	*n*	Mean ± SD	
Intellectual functioning^1^					
cVIQ	69	41.5 *±* 23.2	18	59.6 *±* 19.7	*0.032*
cPIQ	69	42.1 *±* 20.3	19	54.1 *±* 14.1	0.138
cWMI	25	30.0 *±* 11.4	9	51.7 *±* 13.0	0.075
cPSI	34	38.9 *±* 11.6	12	62.8 *±* 20.0	0.004*
cFSIQ	69	28.9 *±* 23.8	18	49.9 *±* 18.1	0.019*
ASD features^2^					
ADOS CSS	64	6.67 *±* 2.06	16	6.00 *±* 2.31	0.930
SA CSS	64	6.38 *±* 2.26	16	5.69 *±* 2.18	0.658
RRB CSS	64	7.64 *±* 1.92	16	7.31 *±* 1.78	0.710
Maladaptive behaviours^3^					
Irritability	70	15.5 *±* 11.4	20	9.00 *±* 7.58	0.011*
Lethargy	70	6.71 *±* 5.42	20	4.65 *±* 3.63	0.117
Stereotypy	70	5.60 *±* 4.66	20	3.90 *±* 4.39	0.233
Hyperactivity	70	12.1 ± 8.28	20	8.35 ± 7.63	0.071
Inappropriate speech	70	4.90 ± 3.50	20	2.80 ± 3.27	0.009*
Social avoidance	70	3.47 ± 2.75	20	2.05 ± 3.07	0.012*
ABC total	70	48.3 ± 28.3	20	30.8 ± 23.4	0.012*
ABC UI	70	0.62 ± 0.19	20	0.73 ± 0.16	0.006*

^1^Semi-parametric regression adjusted for country, age and ADOS CSS

^2^Robust regression adjusted for cFSIQ

^3^Robust regression adjusted for age

*p value remained < 0.05 after adjustment for multiple testing using FDR

There were no significant differences between the proportion of participants with FM (89.2%) and PM/FM mosaicism (94.4%) who met criteria for ASD (*p* = 0.668). The two groups also did not significantly differ on each of the ADOS-2 CSS, both prior to and after adjusting for cFSIQ (Table 4). Similar results for ADOS-2 CSS were observed for both stratified age groups (Additional file 1: Tables S12 and S13).

The PM/FM mosaic group had significantly lower scores on the irritability, inappropriate speech and social avoidance subscales of the ABC-C compared with the FM-only group, after controlling for age. The PM/FM mosaic group also had lower total ABC-C scores and a higher UI (Table 4). No other significant differences were observed between the two groups on the ABC-C. The proportion of males falling into each of the UI categories did not significantly differ between males with FM-only and males with PM/FM mosaicism (Fisher’s exact test, *p* = 0.292). When stratified by age, no significant differences were observed for males under 13 years after adjusting for multiple testing (Additional file 1: Table S12). However, for those males aged 13 and over, all ABC-C domains significantly differed between the PM/FM mosaic and FM-only groups, except hyperactivity (Additional file 1: Table S13).

### Comparison of intellectual functioning, ASD features and maladaptive behaviours between females with FM-only and PM/FM mosaicism

A significantly greater proportion of females with FM-only had FSIQ < 70 (57.1%) compared with females with PM/FM mosaicism (12.5%; *p* = 0.044). Females with PM/FM mosaicism had higher scores on all corrected intellectual functioning domains compared with females with FM-only (Table 5). These same findings were shown when using standard IQ scores and when the MSEL and WISC-III were removed from corrected scores (Additional file 1: Table S14 and S15, respectively).

**Table 5:** Comparison of females by allelic class on corrected intellectual functioning, ASD features and maladaptive behaviours

	FM-only		PM/FM Mosaic		
	*n*	Mean ± SD	*n*	Mean ± SD	*p*
Intellectual functioning^1^					
cVIQ	28	70.7 ***±*** 17.0	8	85.9 ***±*** 20.0	0.024*
cPIQ	28	66.2 ***±*** 17.2	8	78.8 ***±*** 12.3	0.006*
cWMI	9	59.8 ***±*** 17.2	4	80.0 ***±*** 15.0	0.021*
cPSI	16	75.3 ***±*** 19.0	6	92.7 ***±*** 18.1	0.014*
cFSIQ	28	64.5 ***±*** 17.3	8	79.4 ***±*** 15.2	0.019*
ASD features^2^					
ADOS CSS	24	4.75 ***±*** 2.09	8	3.88 ***±*** 2.85	0.806
SA CSS	24	4.71 ***±*** 2.03	8	4.13 ***±*** 2.59	0.664
RRB CSS	24	6.67 ***±*** 2.30	8	4.63 ***±*** 3.16	0.523
Maladaptive behaviours^2^					
Irritability	27	11.8 ***±*** 12.0	7	3.57 ***±*** 3.51	0.036*
Lethargy	27	5.44 ***±*** 5.73	7	1.43 ***±*** 1.90	0.037*
Stereotypy	27	3.48 ***±*** 4.58	7	0.00 ***±*** 0.00	NA
Hyperactivity	27	8.33 ***±*** 7.69	7	0.86 ***±*** 0.90	< 0.001*
Inappropriate speech	27	3.19 ***±*** 3.04	7	0.29 ***±*** 0.49	0.001*
Social avoidance	27	2.67 ***±*** 3.34	7	1.29 ***±*** 2.56	0.429
ABC Total	27	34.9 ***±*** 30.3	7	7.43 ***±*** 6.40	0.001*
ABC UI	27	0.69 ***±*** 0.21	7	0.88 ***±*** 0.06	0.005*

^1^Robust regression adjusted for ADOS CSS

^2^Robust regression adjusted for cFSIQ

*p value remained < 0.05 after adjustment for multiple testing using FDR

Seventeen (70.8%) females with FM-only met criteria for ASD on the ADOS-2 compared with four (50%) in the PM/FM mosaic group. However, this difference was not significant (Fisher’s exact test, *p* = 0.397). Furthermore, females with FM-only and PM/FM mosaicism did not differ significantly on any of the ADOS-2 CSS (Table 5).

Females with FM-only had significantly higher scores on the irritability, lethargy, hyperactivity and inappropriate speech subscales of the ABC-C, in addition to significantly higher overall scores and a significantly lower UI (Table 5), after adjusting for cFSIQ. All seven (100.0%) females with PM/FM mosaicism fell in the very high or high UI category compared to twelve (44.4%) females with FM-only; this difference was significant (Fisher’s exact test, *p* = 0.046). Of note, the female cohorts could not be stratified by age given the small number of females in the 13 and over age group.

## Discussion

This study contributes to the field by providing a neurodevelopmental characterisation (ID, ASD and maladaptive behaviours) in individuals with FXS, stratified by sex and presence/absence of mosaicism. To the best of the authors’ knowledge, this is the first study to compare these features between females with FM-only and with PM/FM mosaicism. Using a large international cohort of individuals with FXS, females were shown to have a milder clinical phenotype in comparison with males, with this attenuation in phenotype being more pronounced in females with PM/FM mosaicism. Similarly, males with PM/FM mosaicism also presented with a milder phenotype in comparison with their FM-only counterparts. The differences observed between sexes and allelic classes are primarily a result of the more severe cognitive impairment in males and FM-only individuals, respectively. These results are likely underpinned by *FMR1*-specific molecular differences between sex and allelic classes. Specifically, *FMR1* promoter methylation mosaicism (reviewed in Kraan et al. [68]) has been inversely correlated with FMRP expression in blood and IQ scores in individuals with FXS of both sexes [60, 69, 70].

### Intellectual functioning

Consistent with previous literature [13, 71], approximately 90% of males had FSIQ < 70 compared with 47% of females. Males also had significantly lower corrected IQ scores on all domains compared with females, which remained significant when stratified by age. Significant variability was observed within both the male and female cohorts as reflected in the range of standard IQ scores (Additional file 1: Table S4).

Although males with FM-only and PM/FM mosaicism did not differ on the proportion having FSIQ < 70, males with PM/FM mosaicism presented with higher corrected IQ scores on all domains though only cPSI and cFSIQ reached statistical significance. When using standard IQ scores VIQ, WMI and PSI were all significantly higher in the PM/FM mosaic group. Closer inspection of the data stratified by age revealed these significant differences were also observed in the younger age group (< 13 years), but not in the older age group (≥ 13 years), likely due to the small sample size of PM/FM mosaic males (*n* = 6) in the older age bracket. Thus, while the majority of males still had IQ scores in the extremely low range, males with PM/FM mosaicism tended to have IQ scores within the milder ID range. The subtle differences that were observed between these two groups (FM-only versus PM/FM mosaic) when comparing index scores demonstrate the significant impact the floor effect has on exploring the phenotype in males with FXS, though there was less impact in females with FXS. Females with PM/FM mosaicism were less likely to have FSIQ < 70 compared with females with FM-only, and all corrected IQ scores were significantly higher in the PM/FM mosaic group.

### ASD features

Males were more likely to meet criteria for ASD on the ADOS-2 compared with females, though within each sex, the proportion of FM-only and PM/FM mosaic cases meeting ADOS-2 criteria did not significantly differ. When using gold standard diagnostic instruments (i.e. ADOS, ADI-R and DSM criteria) as many as 60% of males with FXS presented with behaviours that supported a comorbid diagnosis of ASD [72]. Thus, the proportion of those meeting criteria for ASD in the current study (males 90.4% and females 65.6%) are somewhat higher than previous estimates, though are similar to reports on the prevalence of ASD symptoms more generally in children with FXS (approximately 90% of males and a third of females) [11, 25]. Nonetheless, the proportion meeting ADOS-2 criteria for ASD is not an accurate reflection of the proportion of individuals with FXS who would meet DSM-5 criteria for ASD.

Significant discordance has been observed even between gold standard diagnostic assessments when determining the presence of ASD in those with FXS. In particular, one study demonstrated good agreement between ADOS-G and DSM-IV criteria [72], while another found a high false positive rate using the ADOS-G compared with ADI-R and DSM-IV criteria combined [20]. The revised diagnostic algorithms of the ADOS-2 and changes to ASD diagnostic criteria within the DSM-5 complicate this picture further. The diagnostic algorithm of the ADOS-G did not incorporate repetitive and restricted behaviours and given that these types of behaviours, particularly hand flapping and perseveration of speech, are common in those with FXS [73], the ADOS-2 may increase rates of ASD in FXS in comparison with ADOS-G. Moreover, a recent longitudinal study of ASD in FXS demonstrated increased rates of ASD over time, with 80% of males (*M*_age_ = 11.50 years) and 41.7% of females (*M*_age_ = 11.21 years) meeting criteria for ASD at time 2 using the ADOS-2 algorithms [24]. Similarly, a non-significant trend for social affect CSS scores to increase with chronological age was observed in another longitudinal study of males with FXS [27]. Thus, the use of the ADOS-2 in addition to the wide age range of participants in the current study may explain the elevated rates and features associated with ASD.

Thurman and colleagues [27] also demonstrated that increases in nonverbal ability were associated with decreases in SA CSS and RRB CSS in their longitudinal study of males with FXS (FM-only + PM/FM mosaic). We also found significant associations between nonverbal IQ (cPIQ) and both SA and RBB CSS in males with FXS, and between cPIQ and RRB CSS in females. It has been suggested that individuals with better nonverbal abilities may have a greater range of interests and activities, and may also be more able to engage in more productive rather than repetitive behaviours compared with children with poorer nonverbal abilities [27, 74]. Expressive language skills were also associated with less repetitive behaviours in males with FXS [27]. However, in the current study, associations with cVIQ and RRB CSS were only found in the female cohort. The very low verbal ability scores obtained using the Wechsler scales to assess VIQ may explain the lack of associations in our male cohort.

No differences were observed between sexes on ADOS-2 CSS after controlling for cFSIQ. Thus, the increased prevalence of features associated with ASD in males with FXS compared with females is likely due to the greater severity of intellectual impairment. In line with this, when we adjusted for ADOS CSS for comparisons of intellectual functioning, the differences between males and females remained significant. Nonetheless, differences were obtained at an item level between males and females, particularly the social interaction items. Females were more likely to effectively use eye contact, initiate joint attention, respond to their name, share enjoyment and show objects and toys of interest to the examiner/caregiver. Moreover, females more frequently attempted to engage the examiner (e.g. amount of social overtures) and their caregivers and displayed more creative play skills in comparison with males. More specifically, only a very small proportion of females (6.3%) showed atypical amount of social overtures to the caregiver and for most females, initiation of joint attention and amount of reciprocal communication was typical. Yet, approximately 75% of females presented with atypical *quality* of social overtures. This is consistent with the view that individuals with FXS are interested in engaging socially with others, though other factors such as anxiety and hyperarousal may interfere with the quality of these social interactions [75]. As described by Mazzocco and colleagues [76], language abilities can have negative impacts on social interactions, even in brief encounters with an unfamiliar adult. Specifically, girls with FXS (*n* = 20; VIQ > 70) asked fewer questions overall, fewer questions that logically flowed from the previous utterance, and spoke more repetitious phrases compared with typically developing peers; factors that would contribute to poorer quality of social overtures in an ADOS-2 assessment. Turkstra and colleagues [77] also demonstrated that the gap in performance on social cognition tasks for females with FXS compared with typically developing peers is likely attributed to general cognitive functions such as language and IQ. This phenomenon is likely further inflated in males with FXS, given their more impaired verbal abilities. Interventions that target the contributions of cognitive and language deficits may assist with social interaction skills.

Interestingly, the proportion of males and females presenting with repetitive and restricted behaviours only differed at the item level for unusual sensory interests, with more males engaging in such behaviours compared with females. The lack of differences on repetitive behaviours, as well as hand mannerisms is not surprising given that these are typical features that are associated with FXS in both males [78] and females [17, 18]. In particular, preoccupations with one particular or parts of object(s), distress due to environmental changes and a restricted range of interests have been reported for both males and females with FXS [17]. These item-level differences remained significantly different between males and females for the younger age group (< 13 years), but not the older age group (≥ 13 years); this is likely due to the small sample of females in the older age group. Increased rates of RRBs can further impede daily functioning by creating a barrier to learning and social interaction [79]. Thus, interventions that aim to alleviate these behaviours would also be beneficial. However, identification of the mechanism for the specific RRB would need to be targeted (e.g. cognitive ability, anxiety), and this likely varies for each individual [78].

These findings are in contrast to a study comparing males and females with idiopathic ASD [33], where females who met criteria for ASD on a ‘gold standard’ measure (i.e. ADOS-2 or ADI-R) had similar profiles to males on the ADOS-2 after adjusting for multiple comparisons. However, females were reported by their parents to have greater autistic traits compared to males on the Social Responsiveness Scale (SRS) [80]. This may be due to the fact that the SRS provides norms for males and females separately, while the ADOS-2 is normed predominantly on a sample of males with idiopathic ASD. To further elucidate the autistic profile in females with FXS, the use of other assessment tools such as the SRS may be beneficial.

No differences were observed between FM-only and PM/FM mosaic cases on any ADOS CSS in either sex, after adjusting for cFSIQ. Other researchers investigating the associations between FXS-specific molecular variables and ASD phenomenology reported that FMRP did not account for unique variance in predicting ASD symptom severity over and above the contribution of nonverbal IQ [81]. However, in our recent study on a sub-sample of those individuals included in the current study, we demonstrated that those FM-only males who had incomplete silencing of *FMR1* mRNA FM alleles had greater autistic features based on the ADOS CSS [39]. This suggested that overexpression of expanded FM mRNA may contribute towards the ASD features seen in this sub-group of males with FXS. However, these findings need replication in larger independent cohorts.

### Maladaptive behaviours

Although maladaptive behaviours did not significantly differ between males and females after adjusting for cFSIQ, significant differences were observed between FM-only and PM/FM mosaic cases within each of the sexes. In particular, males with FM-only were reported by their caregivers to be more irritable, use more inappropriate speech and to be more socially avoidant compared with males with PM/FM mosaicism. Moreover, the quality of life was reported to be significantly poorer in FM-only males. When stratified by age, no significant differences were observed between these two groups for the under 13 age group. While significant differences were observed for the older age group on all the ABC-C subscales, except hyperactivity. However, these findings should be interpreted with caution given only six males with PM/FM mosaicism wereincluded in the analysis.

Similarly, females with FM-only were reported to be more irritable, lethargic and hyperactive and to use more inappropriate speech compared with their PM/FM mosaic counterparts. However, the two female groups did not differ on the social avoidance subscale which is consistent with the more social approach type behaviours that were observed in the ADOS-2. Of note, all females with PM/FM mosaicism were reported to have high or very high quality of life compared with only 44% of those in the female FM-only group. Thus, parent reports of behaviours appear to significantly differ between allelic classes despite no significant differences being observed on the ADOS-2. Unlike the ADOS-2 administrators/coders, parents are not blinded to allelic classification and consequently may be biased in their responses on the ABC-C given the prognostic information they are given at the time of diagnosis.

### Limitations

An important limitation of the current study is the use of several assessment types for intellectual functioning. Specifically, the WISC-III Chilean edition is not directly comparable with the other Wechsler scales used in this study. Further, while the MSEL has demonstrated good convergent validity with other intellectual functioning measures in a sample of children with ASD [59], the MSEL may overestimate cognitive abilities in comparison with the Wechsler scales, particularly in children with FXS. Behavioural challenges that emerge later in childhood including inattention and hyperactivity may impact the child’s ability to complete more structured intellectual functioning assessments such as the Wechsler scales.

Evidence for the influence of these assessments is demonstrated when significant differences between allelic classifications within the male cohort were lost when the MSEL and WISC-III were removed. Within the WISC-III Coding and Arithmetic are included in the performance and verbal indexes, respectively. Coding assesses processing speed skills while Arithmeticassesses working memory. Working memory and processing speed tasks can be particularly difficult for males with FXS. The significant flooring associated with males with FXS has a clear impact on scores and thus the understanding of the variability in the phenotype. Thus, there is still a great need to develop an appropriate and accurate assessment battery that can be used longitudinally and that is inclusive for all cognitive abilities. Such an assessment battery will assist in understanding developmental trajectories of individuals with FXS but will also be extremely beneficial for clinical trials. While there is good convergent validity between each of Wechsler scales according to the test manuals [54–56], this might not hold for individuals with ID. Thus, the development and use of a single intellectual functioning assessment tool that is sensitive to variation of cognitive skills in individuals of a wide range, from infancy to adulthood, will be beneficial for future research and clinical practice.

Another limitation to the current study is the wide age range of the participants (1–43 years). While age was used as a covariate (where appropriate) and analyses were also conducted with the groups stratified into two age groups, the small number of females in the older age group prevented further stratification into three categories: (i) childhood (< 13 years); (ii) adolescence (≥ 13 years and ≤ 18 years); (iii) adulthood (19+ years). Future research with larger samples of females would be beneficial. Moreover, detection of ASD features may be more difficult to detect in those aged under 2 years. Nonetheless, for those children in the current study, four children were aged under 2 years and completed a Toddler module of the ADOS-2. All these children met the requirements of this module, cruising/walking and based on the MSEL, a nonverbal mental age greater than 12 months [82]. Two of these children fell in the mild-to-moderate range of concern, one in the moderate-to-severe range of concern and one in the little-to-no concern range. Suggesting that ASD features were accurately detected for participants < 2 years of age.

Lastly, this is not a population-based study and recruitment was consequently confounded by site of recruitment. There is a likely ascertainment bias, particularly in the female group, in which more severe females were involved in the study. This may contribute towards the significant differences that were observed between FM-only and PM/FM mosaic cases. This is an important issue for the broader FXS field and is not unique to this study. Further, obtaining large samples of individuals with PM/FM mosaicism is difficult, as evidenced by this study and other previous studies including males with mosaicism. The small sample of individuals with PM/FM mosaicism in the current study likely reduced statistical power, particularly when groups were stratified by age. Further studies in unbiased and larger samples of females with FM-only and PM/FM mosaicism are needed.

## Conclusions

This study contributes to the field by providing a neurodevelopmental characterisation (ID, ASD and maladaptive behaviours) in individuals with FXS stratified by sex and presence/absence of mosaicism. To the best of the authors’ knowledge, there is no study to date that has compared females with FM-only and females with PM/FM mosaicism on multiple psychological domains using standardised measures. Moreover, there is a lack of research that has compared specific behaviours associated with ASD between males and females. Of note, the findings regarding the differences between males and females on maladaptive behaviours were lost after controlling for IQ, which has not previously been explored.

This study demonstrates that while males with FXS had significantly lower intellectual functioning scores than females with FXS, no significant differences were observed on ASD features after controlling for cFSIQ. This finding suggests that it is primarily the significant cognitive impairment in males with FXS that lead to the increased prevalence of ASD features in comparison with females. Overall ASD features were also shown to be significantly and negatively correlated with cFSIQ scores, in each sex. Taken together, these findings are consistent with the theory that the degree of cognitive impairments in genetic conditions account for the ASD phenomenology via reduced intellectual abilities [83], possibly due to common biochemical and neural dysfunctions leading to both these phenotypic characteristics. These findings have implications in genetic counselling and clinical settings. Nonetheless, the contribution of masking, particularly in higher functioning females with FXS, should be considered and assessments with other ASD assessment tools, which provide norms for each sex may assist to elucidate symptomatology in females.

The ABC-C is widely used as a primary outcome measure in clinical trials in FXS. Given the significant differences seen between the ADOS-2 and ABC-C between allelic classes, caution is advised for the ABC-C as a primary outcome measure when used in isolation. Moreover, current behavioural interventions and treatments for FXS are primarily those that are recommended for individuals with idiopathic ASD and consequently, there is a clear unmet need for treatments that are specific to FXS. Based on the findings of the current study, interventions that target cognitive abilities, particularly nonverbal skills, may in turn reduce the severity of ASD features and other maladaptive behaviours. Reductions in these behaviours have the potential to lead to better outcomes for the affected children and their families.

## Supplementary information

**Additional file 1.** Note S1–S4 and Table S1–S15 Clinical Phenotype of FXS.

## Data Availability

The datasets used and/or analysed during the current study are available from the corresponding author on reasonable request.

## Abbreviations

ABC-C: Aberrant Behavior Checklist-Community
ADI-R: Autism Diagnostic Interview-Revised
ADOS-G: Autism Diagnostic Observation Schedule-Generic
ADOS-2: Autism Diagnostic Observation Schedule-2nd Edition
ADOS CSS: Overall ADOS-2 Calibrated Severity Score
ASD: Autism spectrum disorder
cFSIQ: Corrected Full Scale IQ
cPIQ: Corrected performance IQ
cPSI: Corrected processing speed index
CSS: Calibrated severity score
cVIQ: Corrected verbal IQ
cWMI: Corrected working memory index
DSM-5: Diagnostic and Statistical Manual of Mental Health Disorders-5th Edition
DSM-IV: Diagnostic and Statistical Manual of Mental Health Disorders-4th Edition
FDR: False discovery rate
FM: Full mutation
FMRP: Fragile X mental retardation protein
FSIQ: Full Scale IQ
FXS: Fragile X syndrome
MSEL: Mullen Scales of Early Learning
PIQ: Performance IQ
PM: Pre-mutation
PM/FM: Pre-mutation/full mutation mosaic
PSI: Processing speed index
RRB CSS: Repetitive and Restricted Behaviour Calibrated Severity Scores
SA CSS: Social Affect Calibrated Severity Scores
VIQ: Verbal IQ
WAIS-IV: Wechsler Adult Intelligence Scale-Fourth Edition
WISC-III: Wechsler Intelligence Scale for Children-Third Edition
WISC-IV: Wechsler Intelligence Scale for Children-Fourth Edition
WMI: Working memory index
WPPSI-III: Wechsler Preschool and Primary Scale of Intelligence-Third Edition
