# Supplementary material for: Intellectual functioning and behavioural features associated with mosaicism in fragile X syndrome
Source: J Neurodev Disord. 2019 Dec 26;11:41. doi: 10.1186/s11689-019-9288-7 (PMC6933737; doi:10.1186/s11689-019-9288-7)
Supplement: Supplementary file 1 — Additional file 1. Note S1–S4 and Table S1–S15 Clinical Phenotype of FXS. [file 11689_2019_9288_MOESM1_ESM.docx]

**Note S1.**

Regarding cognitive assessments, one FM male refused to complete the tasks, while two PM/FM mosaic males were administered a cognitive assessment one of these individuals could only obtain a valid PRI and the other could not obtain valid scores on any of the indexes.

**Note S2.**

Four FM females, 5 FM males and 2 PM/FM mosaic males did not complete an ADOS assessment as no research reliable assessor was available for the assessment. The proportion of missing ADOS assessments by sex and allelic classification, is reported in Table S1 with the breakdown of ADOS modules. For all administered ADOS assessments, administrators and coders had completed ADOS-2 for research training and had demonstrated a minimum of 80% reliability with a certified ADOS-2 trainer. A proportion (~12%) of Australian video recordings were recoded for reliability purposes by a certified ADOS-2 trainer (EKB) which demonstrated 81.32% reliability between coders. Similarly, a proportion (~11%) of the Chilean video recordings were re-coded by two individuals who had also completed ADOS-2 research training and were fluent in both Spanish and English. Reliability for Spanish videos was 81.53%.

For the Australian cohort, 79.5% (85.2% of females and 76.1% of males) of participants were assessed by the same examiner for the IQ and ADOS-2 assessments, the remaining participants had the ADOS-2 and IQ assessments completed by two different examiners. In Chile all assessments were undertaken by the same examiner.

**Note S3.**

For all individuals included in the current study it was either the parents or primary caregiver who completed the ABC-C. In Chile, the ABC-C was administered by the assessor to the parents/caregivers, while in Australia the parents/caregivers completed the questionnaire independently at the time of assessment; however the assessor assisted parents when required. Of the 32 individuals aged 18 or above who were involved in the study, 26 were from Chile. For the remaining six Australian adults three were male and their fathers completed the questionnaire together with the mother. For the females two attended the appointment on their own and the ABC-C was not completed and for the third the mother completed the ABC-C but was deemed cognitively able by the first author to complete the questionnaires independently.

**Note S4.**

Given that age was non-linearly associated with corrected intellectual functioning scores in males, the comparison of the difference for each corrected intellectual functioning score between sex or allelic class (in males) was conducted using semi-parametric regression, adjusted for age as a non-parametric component and country and ADOS CSS as parametric component. In females, as there was no relationship between each corrected intellectual functioning score and age, the comparison of the difference for each corrected intellectual functioning score between allelic class, adjusted for ADOS CSS if significant, were carried out using ordinary regression or robust regression (to down-weight the effect of outliers if present).

**Table S1**: Intellectual functioning assessments and ADOS-2 Modules completed by Sex and Allelic Class

|  | **Males** | | **Females** | |
| --- | --- | --- | --- | --- |
|  | *FM*  (*n* = 70) | *PM/FM*  (*n* = 20) | *FM*  (*n* = 28) | *PM/FM*  (*n* = 8) |
| **Intellectual Functioning Assessment** | | |  |  |
| MSEL | 10.0% | 10.0% | 21.4% | 0.0% |
| WPPSI-III | 28.6% | 30.0% | 39.3% | 25.0% |
| WISC-III/IV | 38.6% | 25.0% | 25.0% | 25.0% |
| WAIS-IV | 21.4% | 25.0% | 14.3% | 50.0% |
| No assessment | 1.4% | 10.0% | 0.0% | 0.0% |
| **ADOS-2** |  |  |  |  |
| Toddler Module | 7.1% | 10.0% | 10.7% | 0.0% |
| Module 1 | 42.9% | 25.0% | 21.4% | 0.0% |
| Module 2 | 18.6% | 35.0% | 21.4% | 25.0% |
| Module 3 | 8.6% | 5.0% | 25.0% | 25.0% |
| Module 4 | 15.7% | 15.0% | 7.1% | 50.0% |
| No assessment | 7.1% | 10.0% | 14.3% | 0.0% |

**Table S2**: Proportion of Males and Females, by Allelic Class, with an Invalid or Floor Score on each of the Intellectual Functioning Domains

|  | **Males** | | | | **Females** | | | |
| --- | --- | --- | --- | --- | --- | --- | --- | --- |
|  | *FM-Only* | | *PM/FM Mosaic* | | *FM-Only* | | *PM/FM Mosaic* | |
|  | Invalid  score | Floor  Score | Invalid  score | Floor  Score | Invalid  score | Floor  Score | Invalid  score | Floor  Score |
| VIQ | 29% | 17.1% | 21.1% | 10.0% | 7.1% | 0% | 0% | 0% |
| PIQ | 29% | 25.7% | 15.8% | 5.0% | 3.6% | 0% | 0% | 0% |
| WMI | 12% | 30.0% | 0.0% | 10.0% | 0% | 10.7% | 0% | 0% |
| PSI | 48.6% | 20.0% | 38.5% | 5.0% | 17.9% | 3.6% | 0% | 0% |
| FSIQ | 44.9% | 22.9% | 31.6% | 5.0% | 7.1% | 0.0% | 0% | 0% |

**Table S3**: Correlation (*Corr*) between autism symptoms and corrected intellectual functioning scores.

|  | **Males** | | |  | **Females** | | |
| --- | --- | --- | --- | --- | --- | --- | --- |
|  | *n* | *Corr* | *p* |  | *n* | *Corr* | *p* |
| **SA CSS** |  |  |  |  |  |  |  |
| cVIQ | 80 | -0.303 | **0.006** |  | 32 | -0.364 | **0.041** |
| cPIQ | 81 | -0.419 | **0.0001** |  | 32 | -0.270 | 0.135 |
| **RRB CSS** | |  |  |  |  |  |  |
| cVIQ | 80 | -0.216 | 0.054 |  | 32 | -0.436 | **0.013** |
| cPIQ | 81 | -0.312 | **0.004** |  | 32 | -0.442 | **0.011** |

Correlation computed using Spearman’s correlation, with males cVIQ and cPIQ adjusted for country and age, and unadjusted for females.

**Table S4:** Comparison between males and females on standard intellectual functioning variables

|  | **Males** | | **Females** | |  |
| --- | --- | --- | --- | --- | --- |
|  | *n* | *Mean* ± *SD*  (Range) | *n* | *Mean* ± *SD*  (Range) | *p* |
| VIQ | 63 | 58.5 ± 12.4  43 – 87 | 34 | 76.5 ± 17.0  47 – 122 | **<0.0001** |
| PIQ | 65 | 55.8 ± 10.7  45 – 86 | 35 | 71.0 ± 14.2  49 – 105 | **<0.0001** |
| WMI | 31 | 52.2 ± 4.64  50 – 69 | 13 | 67.8 ± 16.2  50 – 95 | **0.001** |
| PSI | 27 | 56.1 ± 11.2  50 – 97 | 20 | 82.3 ± 18.3  50 – 120 | **<0.0001** |
| FSIQ | 51 | 50.8 ± 11.9  40 – 83 | 34 | 70.7 ± 15.3  41 – 108 | **<0.0001** |

Robust regression was used to compare the differences between sexes, adjusted for ADOS CSS. All p-values < 0.05 after adjustment for multiple testing using FDR.

**Table S5** Comparison of males and females on corrected intellectual functioning variables (MSEL and WISC-III removed).

|  | **Males** | |  | **Females** | |  |
| --- | --- | --- | --- | --- | --- | --- |
|  | *n* | *Mean ± SD* |  | *n* | *Mean ± SD* | *p* |
| cVIQ | 60 | 55.3 ± 15.0 |  | 26 | 73.7 ± 18.5 | **<0.0001** |
| cPIQ | 60 | 50.7 ± 14.2 |  | 26 | 67.5 ± 17.4 | **<0.0001** |
| cFSIQ | 60 | 40.9 ± 18.3 |  | 26 | 66.5 ± 18.6 | **<0.0001** |

*Note.* WMI and PSI are not included in the WISC-III (Chile) or the MSEL, therefore results remain the same as presented in the main text. Semi-parametric regression was used to compare between gender, adjusted for country, age and ADOS CSS. All p-values < 0.05 after adjustment for multiple testing using FDR.

**Table S6:** Comparison between males and females on corrected intellectual functioning scores, autism features, and maladaptive behaviours for individuals aged under 13 years.

|  | Males | | Females | |  |
| --- | --- | --- | --- | --- | --- |
|  | n | Mean ± SD | n | Mean ± SD | p |
| **Intellectual Functioning^1^** |  |  |  |  |  |
| cVIQ | 56 | 45.7 ± 22.8 | 25 | 70.0 ± 17.1 | **0.035*** |
| cPIQ | 57 | 47.1 ± 19.6 | 25 | 66.2 ± 16.7 | **0.020*** |
| cWMI | 8 | 42.6 ± 11.1 | 4 | 54.3 ± 9.39 | 0.094 |
| cPSI | 20 | 55.1 ± 16.5 | 13 | 76.4 ± 22.3 | **0.002*** |
| cFSIQ | 56 | 38.9 ± 23.5 | 25 | 65.1 ± 16.8 | **0.009*** |
| **Autism Features^2^** |  |  |  |  |  |
| ADOS CSS | 56 | 6.29 ± 2.18 | 24 | 4.25 ± 1.94 | 0.071 |
| SA CSS | 56 | 5.96 ± 2.38 | 24 | 4.21 ± 1.77 | 0.608 |
| RRB CSS | 56 | 7.63 ± 1.91 | 24 | 6.21 ± 2.36 | 0.151 |
| **Maladaptive Behaviours^3^** |  |  |  |  |  |
| Irritability | 56 | 16.3 ± 12.1 | 25 | 11.6 ± 12.3 | 0.694 |
| Lethargy | 56 | 6.95 ± 5.90 | 25 | 5.24 ± 6.06 | 0.912 |
| Stereotypy | 56 | 6.32 ± 4.89 | 25 | 3.44 ± 4.79 | 0.052 |
| Hyperactivity | 56 | 13.9 ± 8.55 | 25 | 7.84 ± 8.19 | 0.078 |
| Inappropriate Speech | 56 | 3.68 ± 3.54 | 25 | 2.92 ± 3.13 | 0.971 |
| Social Avoidance | 56 | 2.82 ± 2.89 | 25 | 2.44 ± 3.54 | 0.261 |
| ABC Total | 56 | 50.0 ± 31.4 | 25 | 33.4 ± 32.0 | 0.430 |
| ABC UI | 56 | 0.63 ± 0.20 | 25 | 0.73 ± 0.21 | 0.423 |

^1^Semi-parametric regression adjusted for country, age and ADOS CSS whenever significant; ^2^Robust regression adjusted for country and cFSIQ; ^3^Robust Regression adjusted for cFSIQ. *p-value remained <0.05 after adjustment for multiple testing using FDR.

**Table S7** Comparison between males and females on corrected intellectual functioning scores, autism features, and maladaptive behaviours for individuals aged 13 and over.

|  | **Males** | | **Females** | |  |
| --- | --- | --- | --- | --- | --- |
|  | *n* | *Mean ± SD* | *n* | *Mean ± SD* | *p* |
| **Intellectual Functioning^1^** |  |  |  |  |  |
| cVIQ | 31 | 44.5 ± 25.3 | 11 | 83.3 ± 19.2 | **<0.0001*** |
| cPIQ | 31 | 40.3 ± 19.4 | 11 | 75.5 ± 16.1 | **<0.0001*** |
| cWMI | 26 | 33.7 ± 15.8 | 9 | 71.2 ± 19.7 | **<0.0001*** |
| cPSI | 26 | 37.5 ± 14.5 | 9 | 85.3 ± 15.8 | **<0.0001*** |
| cFSIQ | 31 | 23.2 ± 22.4 | 11 | 74.1 ± 19.2 | **<0.0001*** |
| **Autism Features^2^** |  |  |  |  |  |
| ADOS CSS | 24 | 7.13 ± 1.85 | 8 | 5.38 ± 3.11 | 0.717 |
| SA CSS | 24 | 6.88 ± 1.80 | 8 | 5.63 ± 2.92 | 0.755 |
| RRB CSS | 24 | 7.46 ± 1.86 | 8 | 6.00 ± 3.55 | 0.715 |
| **Maladaptive Behaviours^3^** |  |  |  |  |  |
| Irritability | 31 | 9.97 ± 6.91 | 9 | 6.00 ± 6.52 | 0.931 |
| Lethargy | 31 | 4.90 ± 3.30 | 9 | 2.89 ± 2.37 | 0.973 |
| Stereotypy | 31 | 3.23 ± 3.45 | 9 | 0.89 ± 1.54 | 0.479 |
| Hyperactivity | 31 | 6.55 ± 4.95 | 9 | 3.89 ± 4.17 | 0.723 |
| Inappropriate Speech | 31 | 5.84 ± 3.05 | 9 | 1.67 ± 2.29 | 0.196 |
| Social Avoidance | 31 | 3.39 ± 2.56 | 9 | 2.22 ± 2.22 | 0.316 |
| ABC Total | 31 | 33.9 ± 16.5 | 9 | 17.6 ± 15.7 | 0.979 |
| ABC UI | 31 | 0.66 ± 0.16 | 9 | 0.74 ± 0.21 | 0.948 |

^1^Semi-parametric regression adjusted for country, age and ADOS CSS whenever significant; ^2^Robust regression adjusted for country and cFSIQ; ^3^Robust Regression adjusted for cFSIQ. *p-value remained <0.05 after adjustment for multiple testing using FDR.

**Table S8:** Comparison between males and females on key ADOS-2 items for individuals aged under 13 years.

|  | **Males** | | **Females** | |  |
| --- | --- | --- | --- | --- | --- |
|  | *n* | % atypical | *n* | % atypical | ***p*** |
| **SA** |  | |  | |  |
| Pointing | 55 | 74.6% | 17 | 76.5% | 0.999 |
| Gestures | 58 | 63.8% | 24 | 41.7% | 0.087 |
| Eye contact | 58 | 81.0% | 24 | 50.0% | **0.007*** |
| Facial Expressions | 58 | 84.5% | 24 | 70.8% | 0.220 |
| Shared Enjoyment | 58 | 51.7% | 24 | 8.33% | **0.001*** |
| Showing | 55 | 78.2% | 17 | 35.3% | **0.002*** |
| Response to Joint Attention | 55 | 32.7% | 17 | 11.8% | 0.125 |
| Initiation of Joint Attention | 55 | 69.1% | 17 | 29.4% | **0.005*** |
| Quality of Social Overtures | 58 | 86.2% | 24 | 79.2% | 0.510 |
| Rapport | 58 | 81.0% | 24 | 54.2% | **0.026** |
| **RRB** |  | |  | |  |
| Sensory | 58 | 67.2% | 24 | 29.2% | **0.003*** |
| Mannerisms | 58 | 65.5% | 24 | 50.0% | 0.220 |
| Repetitive and Stereotyped Behaviours | 58 | 84.5% | 24 | 75.0% | 0.354 |
| Stereotyped Language | 37 | 70.3% | 19 | 63.2% | 0.763 |
| **Other** |  |  |  |  |  |
| Use of Body as Tool | 36 | 36.1% | 9 | 11.1% | 0.236 |
| Response to Name | 55 | 45.5% | 17 | 11.8% | **0.020*** |
| Amount of Social Overtures (Examiner) | 58 | 81.0% | 24 | 37.5% | **<0.001*** |
| Amount of Social Overtures (Caregiver) | 45 | 57.8% | 16 | 6.30% | **<0.001*** |
| Amount of Reciprocal Communication | 20 | 70.0% | 15 | 26.7% | **0.018*** |
| Functional Play | 55 | 72.7% | 17 | 29.4% | **0.003*** |
| Imaginative/Creative Play | 58 | 91.4% | 23 | 60.9% | **0.002*** |
| Self-Injury | 58 | 13.8% | 24 | 0.0% | 0.097 |
| Anxiety | 58 | 37.9% | 23 | 21.7% | 0.198 |

All p-values computed using Fisher’s exact test*; **p-value remained <0.05 after adjusting for multiple testing using FDR.

**Table S9:** Comparison between males and females on key ADOS-2 items for individuals aged 13 years and over.

|  | **Males** | | **Females** | |  |
| --- | --- | --- | --- | --- | --- |
|  | *n* | % atypical | *n* | % atypical | ***p*** |
| **SA** |  | |  | |  |
| Pointing | NA |  | NA |  |  |
| Gestures | 25 | 36.0% | 8 | 25.0% | 0.687 |
| Eye contact | 25 | 84.0% | 8 | 62.5% | 0.320 |
| Facial Expressions | 25 | 96.0% | 8 | 75.0% | 0.139 |
| Shared Enjoyment | 25 | 44.0% | 8 | 50.0% | 0.999 |
| Showing | NA |  | NA |  |  |
| Response to Joint Attention | NA |  | NA |  |  |
| Initiation of Joint Attention | NA |  | NA |  |  |
| Quality of Social Overtures | 25 | 92.0% | 8 | 62.5% | 0.078 |
| Rapport | 25 | 52.0% | 8 | 25.0% | 0.242 |
| **RRB** |  | |  | |  |
| Sensory | 25 | 52.0% | 8 | 37.5% | 0.688 |
| Mannerisms | 25 | 44.0% | 8 | 37.5% | 0.999 |
| Repetitive and Stereotyped Behaviours | 25 | 48.0% | 8 | 50.0% | 0.999 |
| Stereotyped Language | 24 | 91.7% | 8 | 62.5% | 0.085 |
| **Other** |  |  |  |  |  |
| Use of Body as Tool | NA |  | NA |  |  |
| Response to Name | NA |  | NA |  |  |
| Amount of Social Overtures (Examiner) | 25 | 52.0% | 8 | 37.5% | 0.688 |
| Amount of Social Overtures (Caregiver) | NA |  | NA |  |  |
| Amount of Reciprocal Communication | 22 | 63.6% | 8 | 37.5% | 0.242 |
| Functional Play | NA |  | NA |  |  |
| Imaginative/Creative Play | 25 | 100% | 8 | 87.5% | 0.242 |
| Self-Injury | 25 | 8.00% | 8 | 0.00% | 0.999 |
| Anxiety | 25 | 32.0% | 8 | 75.0% | **0.047** |

NA = no data for females; all p-values computed using Fisher’s exact test*;* no p-value remained <0.05 after adjusting for multiple testing using FDR.

**Table S10** Comparison of males by allelic class on standard intellectual functioning variables

|  | **FM-Only** | | **PM/FM Mosaic** | |  |
| --- | --- | --- | --- | --- | --- |
|  | *n* | *Mean ± SD* | *n* | *Mean ± SD* | *p* |
| VIQ^+^ | 48 | 56.0 ± 11.0 | 15 | 66.3 ± 13.7 | **0.024*** |
| PIQ^+^ | 49 | 54.3 ± 10.7 | 16 | 58.4 ± 10.7 | 0.426 |
| WMI^+^ | 22 | 50.2 ± 0.85 | 9 | 57.1 ± 6.73 | **0.010*** |
| PSI^+^ | 19 | 51.4 ± 3.60 | 8 | 67.1 ± 15.4 | **0.007*** |
| FSIQ^#^ | 38 | 48.6 ± 11.1 | 13 | 57.2 ± 12.2 | 0.104 |

^+^Ordinary and ^#^robust regression were used to compare differences between allelic classes adjusted for ADOS CSS; **p*-value <0.05 after adjustment for multiple testing using FDR.

**Table S11** Comparison of males by allelic class on corrected intellectual functioning variables (MSEL and WISC-III removed)

|  | **FM-only** | | **PM/FM Mosaic** | |  |
| --- | --- | --- | --- | --- | --- |
|  | *n* | *Mean* ± *SD* | *n* | *Mean* ± *SD* | *p* |
| cVIQ | 45 | 53.0 ± 14.2 | 15 | 62.1 ± 15.8 | 0.272 |
| cPIQ | 45 | 49.2 ± 14.6 | 15 | 55.4 ± 12.4 | 0.936 |
| cFSIQ | 45 | 37.4 ± 18.2 | 15 | 51.1 ± 14.8 | 0.205 |

*Note.* WMI and PSI are not included in WISC-III or MSEL therefore results remain the same as presented in the main text. Semi-parametric regression was used to compare differences between allelic classes, adjusted for country and age.

**Table S12:** Comparison of males by allelic class on corrected intellectual functioning, autism features, and maladaptive behaviours for individuals aged under 13 years.

|  | **FM-only** | | **PM/FM Mosaic** | |  | |  |
| --- | --- | --- | --- | --- | --- | --- | --- |
|  | *n* | *Mean ± SD* | *n* | *Mean ± SD* | *p* | |  |
| **Intellectual Functioning^1^** | | | | | |  |  |
| cVIQ | 44 | 42.3 *±* 22.5 | 12 | 58.1 *±* 19.9 | **0.034*** | |  |
| cPIQ | 44 | 45.2 *±* 20.7 | 13 | 53.8 *±* 13.9 | 0.133 | |  |
| cWMI | 5 | 35.8 *±* 5.22 | 3 | 54.0 *±* 8.00 | **0.019*** | |  |
| cPSI | 14 | 47.1 *±* 10.5 | 6 | 73.7 *±* 12.5 | **<0.001*** | |  |
| cFSIQ | 44 | 35.3 *±* 23.9 | 12 | 51.9 *±* 17.1 | **0.020*** | |  |
| **Autism Features^2^** |  |  |  |  |  |  |  |
| ADOS CSS | 44 | 6.38 *±* 2.13 | 12 | 5.92 *±* 2.43 | 0.722 | |  |
| SA CSS | 44 | 6.14 *±* 2.40 | 12 | 5.33 *±* 2.31 | 0.760 | |  |
| RRB CSS | 44 | 7.66 *±* 1.99 | 12 | 7.50 *±* 1.68 | 0.999 | |  |
| **Maladaptive Behaviours^3^** | | | | | | | |
| Irritability | 44 | 17.8 *±* 12.6 | 14 | 10.5 *±* 8.33 | **0.040** | |  |
| Lethargy | 44 | 7.39 *±* 6.31 | 14 | 5.71 *±* 3.83 | 0.485 |  |  |
| Stereotypy | 44 | 6.52 *±* 4.99 | 14 | 5.29 *±* 4.55 | 0.429 |  |  |
| Hyperactivity | 44 | 14.8 ± 8.42 | 14 | 9.79 ± 8.27 | **0.044** |  |  |
| Inappropriate Speech | 44 | 3.93 ± 3.52 | 14 | 2.50 ± 3.41 | 0.080 |  |  |
| Social Avoidance | 44 | 3.02 ± 2.82 | 14 | 2.64 ± 3.46 | 0.504 |  |  |
| ABC Total | 44 | 53.4 ± 32.1 | 14 | 36.4 ± 25.2 | 0.095 |  |  |
| ABC UI | 44 | 0.61 ± 0.20 | 14 | 0.69 ± 0.17 | 0.236 |  |  |

^1^Semi-parametric regression adjusted for country, age and ADOS CSS whenever significant; ^2^Robust regression adjusted for cFSIQ; ^3^Robust regression adjusted for age.

*p-value remained <0.05 after adjustment for multiple testing using FDR.

**Table S13:** Comparison of males by allelic class on corrected intellectual functioning, autism features, and maladaptive behaviours for individuals aged 13 years and over.

|  | **FM-only** | | **PM/FM Mosaic** | |  | |  |
| --- | --- | --- | --- | --- | --- | --- | --- |
|  | *n* | *Mean ± SD* | *n* | *Mean ± SD* | *p* | |  |
| **Intellectual Functioning^1^** | | | | | |  |  |
| cVIQ | 25 | 40.1 *±* 24.7 | 6 | 62.7 *±* 20.6 | **0.048** | |  |
| cPIQ | 25 | 36.8 *±* 18.7 | 6 | 54.7 *±* 15.9 | 0.138 | |  |
| cWMI | 20 | 28.6 *±* 12.1 | 6 | 50.5 *±* 15.5 | 0.775 | |  |
| cPSI | 20 | 33.1 *±* 8.54 | 6 | 52.0 *±* 21.0 | 0.833 | |  |
| cFSIQ | 25 | 17.8 *±* 19.5 | 6 | 45.8 *±* 20.8 | **0.034** | |  |
| **Autism Features^2^** |  |  |  |  |  |  |  |
| ADOS CSS | 20 | 7.0 ± 3.0 | 4 | 6.0 ± 3.5 | 0.184 | |  |
| SA CSS | 20 | 7.0 ± 3.0 | 4 | 7.0 ± 2.5 | 0.340 | |  |
| RRB CSS | 20 | 8.0 ± 3.0 | 4 | 6.0 ± 2.5 | 0.128 | |  |
| **Maladaptive Behaviours^2^** | | | | | | | |
| Irritability | 26 | 10 *±* 8.0 | 6 | 5.5 *±* 7.0 | **0.040*** | |  |
| Lethargy | 26 | 5.0 *±* 4.0 | 6 | 2.5 *±* 1.0 | **0.002*** |  |  |
| Stereotypy | 26 | 3.5 *±* 6.0 | 6 | 0.0 *±* 1.0 | **0.015*** |  |  |
| Hyperactivity | 26 | 6.0 ± 7.0 | 6 | 4.0 ± 4.0 | 0.255 |  |  |
| Inappropriate Speech | 26 | 6.5 ± 4.0 | 6 | 3.0 ± 5.0 | **0.042*** |  |  |
| Social Avoidance | 26 | 4.0 ± 3.0 | 6 | 0.0 ± 1.0 | **0.003*** |  |  |
| ABC Total | 26 | 32 ± 25 | 6 | 18 ± 13 | **0.006*** |  |  |
| ABC UI | 26 | 0.7 ± 0.3 | 6 | 0.8 ± 0.1 | **0.005*** |  |  |

^1^Semi-parametric regression adjusted for country, age and ADOS CSS whenever significant; ^2^Summary statistics were presented by median and interquartile and non-parametric Mann-Whitney test was used for comparison; *p-value remained <0.05 after adjustment for multiple testing using FDR.

**Table S14** Comparison of females by allelic class on standard intellectual functioning variables

|  | **FM-Only** | | **PM/FM Mosaic** | |  |
| --- | --- | --- | --- | --- | --- |
|  | *n* | *Mean* (*SD*) | *n* | *Mean* (*SD*) | *p* |
| VIQ | 26 | 73.1 (15.7) | 8 | 87.5 (17.2) | **0.021** |
| PIQ | 27 | 68.7 (14.1) | 8 | 78.8 (12.3) | **0.021** |
| WMI | 9 | 62.4 (14.2) | 4 | 80.0 (15.0) | **0.043** |
| PSI | 14 | 77.8 (17.9) | 6 | 92.7 (18.1) | **0.023** |
| FSIQ | 26 | 67.7 (14.7) | 8 | 80.5 (13.8) | **0.019** |

Robust regression was used to compare differences between allelic classes, adjusted for ADOS CSS. All p-values < 0.05 after adjusting for multiple testing using FDR.

**Table S15** Comparison of females by allelic class on corrected intellectual functioning variables with MSEL and WISC removed.

|  | **FM-only** | | **PM/FM Mosaic** | |  |
| --- | --- | --- | --- | --- | --- |
|  | *n* | *Mean* ± *SD* | *n* | *Mean* ± *SD* | *p* |
| cVIQ | 20 | 67.9 ± 15.2 | 6 | 93.3 ± 15.5 | **<0.001** |
| cPIQ | 20 | 63.7 ± 16.8 | 6 | 80.3 ± 14.0 | **0.001** |
| cFSIQ | 20 | 61.2 ± 16.6 | 6 | 84.2 ± 14.2 | **<0.001** |

*Note.* WMI and PSI not included in WISC or MSEL therefore results remain the same as presented in the main text. Robust regression was used to compare differences between allelic classes, adjusted for ADOS CSS. All p-values < 0.05 after adjusting for multiple testing using FDR.
